# Comparative analysis of salivary IL-4 and CXCL6 dynamics following tooth extraction in diabetic and non-diabetic patients

**DOI:** 10.4317/medoral.28058

**Published:** 2026-03-07

**Authors:** Mohammed Amjed Alsaegh, Yousuf Ibrahim Al Shehhi, Shishir Ram Shetty, Okba Mahmoud, Jayaraj Kodangattil Narayanan, Sudhir Rama Varma

**Affiliations:** 1 Department of Oral and Craniofacial Health Sciences, College of Dental Medicine, University of Sharjah. Sharjah, UAE; 2 Research Institute for Medical and Health Sciences, University of Sharjah. Sharjah, UAE; 3 Department of Clinical Sciences, College of Dentistry, Ajman University, Ajman, UAE; 4 Center for Medical and Bio-allied Health Sciences Research, Ajman University, Ajman, UAE; 5 Basic Medical and Dental Sciences Department, Ajman University, Ajman, UAE

## Abstract

**Background:**

Tooth extraction initiates a regulated inflammatory healing response. Diabetes mellitus may alter this early tissue response; however, data on early salivary cytokine and chemokine changes after extraction in diabetic individuals remain limited. Therefore, this study aimed to compare early postoperative salivary interlukine-4 (IL-4) and CXC motif chemokine ligand 6 (CXCL6) dynamics following tooth extraction in diabetic and non-diabetic individuals.

**Material and Methods:**

A prospective cohort study was conducted including 30 participants, comprising 20 individuals with controlled type 2 diabetes mellitus and 10 healthy controls. Unstimulated whole saliva was collected at three time points: Before extraction, 1 hour post-extraction and 2 days post-extraction. Salivary IL-4 and CXCL6 concentrations were measured using a Luminex multiplex assay. A two-way repeated-measures ANOVA was applied to examine within-subject temporal effects and between-group differences, as well as their interaction.

**Results:**

IL-4 levels did not show significant changes over time (p=0.453) and did not differ between diabetic and non-diabetic participants (p=0.221), with no significant time-by-group interaction (p=0.526). In contrast, CXCL6 demonstrated a significant effect of time (p=0.035) and a significant time-by-group interaction (p=0.043), indicating distinct temporal response patterns between groups, although overall CXCL6 levels did not differ significantly between diabetic and control participants.

**Conclusions:**

Early post-extraction healing in diabetes demonstrates altered inflammatory kinetics. While salivary IL-4 remained stable within the first 48 hours, CXCL6 exhibited significant time and group-dependent modulation, indicating dysregulated early inflammatory responses.

## Introduction

Tooth extraction is a common dental procedure that induces a well-defined wound-healing sequence involving hemostasis, acute inflammation, proliferation, and remodeling. The early phase is characterized by rapid vascular changes and leukocyte infiltration that set the stage for subsequent reparative activity. Proper coordination of these phases is critical for efficient healing, as disruption of early inflammatory kinetics can delay healing or increase the risk of complications (1).

Diabetes mellitus is associated with systemic alterations that compromise normal wound healing. Individuals with diabetes often exhibit prolonged inflammation, impaired re-epithelialization, and altered immune cell function, which contribute to delayed wound closure and increased susceptibility to infections (2). Although clinical glucose testing readily identifies diabetic status, it does not capture how systemic metabolic alterations influence local tissue response dynamics following injury. Understanding early inflammatory mediator behavior may offer mechanistic insight into healing trajectories in diabetic and non-diabetic individuals.

Cytokines and chemokines orchestrate the wound-healing process through tightly regulated, temporally distinct roles. Chemokines, particularly members of the Cys-X-Cys (CXC) family, are crucial for early inflammatory signaling and neutrophil recruitment to the wound site, shaping the initial immune response that precedes tissue repair (3).

Within the cytokine network, interleukin-4 (IL-4) plays a key regulatory role as an anti-inflammatory mediator that promotes macrophage M2 polarization, modulates fibroblast activity, and supports resolution of inflammation and tissue repair (4). IL-4 is also implicated in bone healing through its influence on osteoclast inhibition, making it relevant to the early stages of socket repair (5). Altered IL-4 signaling has been observed in diabetic conditions (6 , 7). However, evidence regarding IL-4 behavior in saliva after dental extraction, particularly in diabetic patients, remains limited. Given that IL-4 is more closely associated with the transition from inflammation to later reparative phases, early post-injury changes may be minimal.

CXC motif chemokine ligand 6 (CXCL6) is a neutrophil-attracting chemokine that participates in early inflammatory signaling through interactions with chemokine receptors such as CXCR1 and CXCR2. Its role in coordinating cell recruitment and early immune responses positions it as a potential indicator of acute tissue response behavior during the initial inflammatory phase (8). Although most research on CXCL6 has focused on chronic diabetic wounds such as foot ulcers, where higher CXCL6 levels correlate with healing outcomes (9). Diabetes is known to impair neutrophil chemotaxis (10 - 12), reduce vascular perfusion (13), and dysregulate chemokine expression (12), suggesting that CXCL6 dynamics may differ in diabetic individuals. Nevertheless, few studies have evaluated CXCL6 levels in saliva in relation to oral wound healing, and its postoperative trajectory in diabetic patients is not well established.

Saliva offers a non-invasive medium for evaluating inflammatory mediators in the oral environment, reflecting both local tissue responses and systemic influences. While previous studies have investigated salivary cytokines in periodontal disease and systemic inflammatory states, data on early temporal changes in salivary mediators following tooth extraction are limited. Moreover, few studies have compared these dynamics between diabetic and non-diabetic individuals using multiple early sampling intervals (14).

Therefore, the present study aimed to evaluate the temporal dynamics of salivary IL-4 and CXCL6 before and after tooth extraction in diabetic and non-diabetic individuals. By sampling at baseline, 1 hour and 2 days post-extraction, this investigation sought to capture early inflammatory response behavior and determine whether diabetes modifies cytokine and chemokine trajectories rather than absolute mediator levels. We hypothesized that IL-4 and CXCL6 exhibit distinct post-extraction temporal expression patterns, and that diabetes modifies the kinetics of these cytokine responses during early socket healing.

## Material and Methods

**Figure 1:**
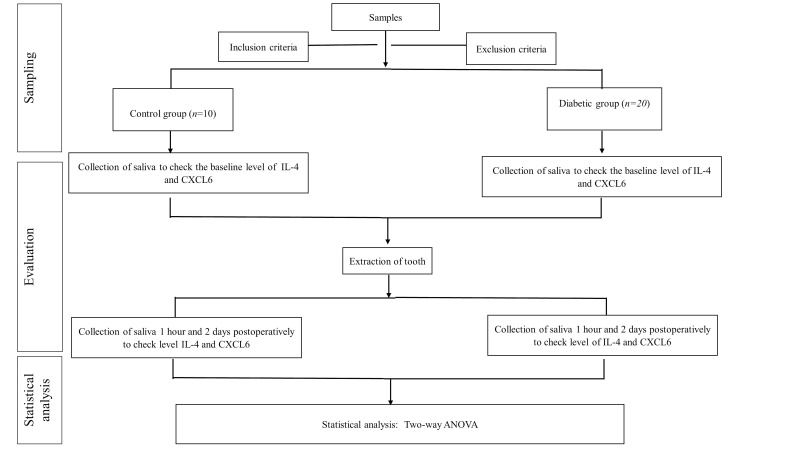
A diagrammatic representation of the chronology of the methodology.

The cohort study design and workflow are illustrated in Figure 1. This prospective observational study included 30 adult patients recruited from the University Dental Hospital of Sharjah. Participants were stratified into a type 2 diabetes mellitus group (n=20) and a healthy control group (n=10). Eligible participants were older than 18 years and required extraction of non-restorable necrotic teeth without periodontal or apical lesions. Patients in the diabetic group had been diagnosed with type 2 diabetes mellitus for at least one year, with HbA1c &lt;7.5% and random glucose &lt;180mg/dL. All were well controlled and had no diabetes-related systemic disease. Controls had no history of systemic illness. Exclusion criteria included tobacco use, immunological disorders, immunosuppressive therapy, recent antibiotic or anti-inflammatory medication, acute dental infections, pregnancy or breastfeeding, need for prophylactic antibiotics, or systemic conditions that could alter inflammatory responses. All participants provided written informed consent. The study complied with the Declaration of Helsinki and was approved by the University of Sharjah Human Research and Ethics Committee (REC-21-06-01-03-S).

1

Sample size calculation was performed using G*Power software (version 3.1.9.7). An a priori power analysis was conducted for a repeated-measures ANOVA with a within-between interaction, corresponding to the planned two-way repeated-measures ANOVA. Assuming a medium effect size (f=0.25), an alpha level of 0.05, a statistical power of 80%, two study groups, three repeated measurements, and an assumed correlation of 0.5 among repeated measures, the required total sample size was estimated to be 28 participants. The present study included 30 participants, thereby exceeding the calculated minimum sample size.

A volume of 5ml of unstimulated whole saliva was collected at three time points: Baseline (pre-extraction), 1 hour post-extraction and 2 days post-extraction. Patients fasted for at least one hour prior to sampling, rinsed with tap water for 2 minutes, and then expectorated into sterile tubes for 5 minutes. Post-extraction samples were collected passively to avoid disturbing clots. All samples were transported on ice and stored at -80 °C until analysis.

Salivary IL-4 and CXCL6 were quantified using a Human Magnetic Pre-Mixed Analyte Luminex Assay (R&amp;D Systems, UK). Samples were thawed, centrifuged at 4000 RCF for 15 min at 4 °C, and diluted 1:1 with calibrant diluent. Standards were prepared by 3-fold serial dilution to generate seven concentrations. Fifty microliters of standards or samples were added in duplicate to a 96-well plate, followed by 50 l of premixed microparticle cocktail. Plates were incubated at room temperature for 2 h on an orbital shaker (800 rpm), washed using a Bio-Plex Washing Station (Bio-Rad Laboratories, USA), incubated with 50 l of biotin-antibody cocktail for 1 h, rewashed, and incubated with 50 l of streptavidin-PE for 30 min. Finally, microparticles were resuspended in 100 l of wash buffer, and fluorescence was measured with a Bio-Plex-200 system (Bio-Rad Laboratories, USA). Data were analyzed using Bio-Plex Manager software. Standard curves were fitted with a 5-parameter logistic regression, and concentrations were expressed as pg/mL and corrected for dilution.

Statistical analyses were performed with SPSS version 28 (IBM Corporation, Armonk, NY, USA). Continuous variables were summarized as means and standard deviations. Two-way repeated-measures ANOVA was used to assess the effects of time (before extraction, 1 hour and 2 days) and group (diabetic vs non-diabetic), and their interaction, on IL-4 and CXCL6 levels. Sphericity was tested using Mauchly's test and corrected with Greenhouse-Geisser adjustment when required. Post-hoc comparisons of estimated marginal means were performed where appropriate, with significance set at p&lt;0.05.

## Results

Participant Characteristics

A total of 30 participants were included, comprising 20 individuals with type 2 diabetes mellitus and 10 healthy controls. The total mean age was 50.37±13.17, and the mean age of the diabetic group was higher than that of the control group (Table 1). All diabetic participants had controlled glycemic status (HbA1c &lt;7.5% and random blood glucose &lt;180 mg/dL). No significant differences were found between groups regarding age or gender distribution (Table 1).

Table 1Salivary IL-4

The mean values of IL-4 in control and diabetic groups during the three-time intervals are shown in Table 2. A two-way repeated-measures ANOVA was performed to assess changes in salivary IL-4 levels over time (before extraction, 1 hour, and 2 days post-extraction) and between diabetic and non-diabetic groups. Mauchly's test indicated that the assumption of sphericity was met (p=0.143); therefore, sphericity-assumed results are reported.

**Figure 2:**
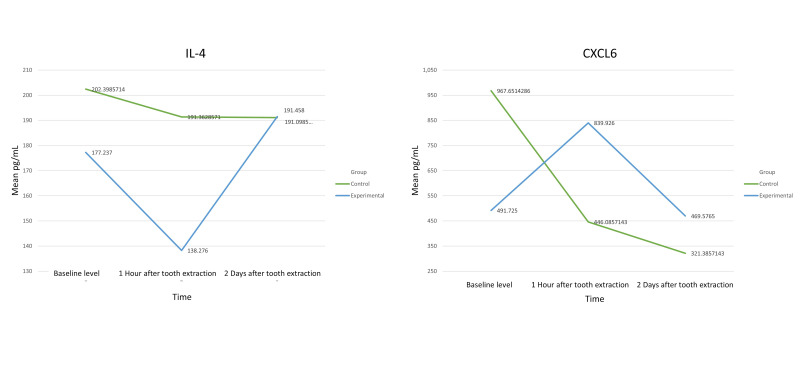
Line graphs illustrating IL-4 and CXCL6 levels at baseline, 1 hour and 48 hours following tooth extraction in individuals with type 2 diabetes mellitus and healthy controls.

There was no significant main effect of time on IL-4 levels (F(2,50)=0.805, p=0.453, partial ²=0.031), indicating that IL-4 concentrations did not change significantly following tooth extraction across the assessed time points. The between-subjects analysis demonstrated no significant difference in overall IL-4 levels between diabetic and non-diabetic participants (F(1, 25)=1.575, p=0.221, partial ²=0.059). In addition, the time-by-group interaction was not significant (F(2, 50)=0.650, p=0.526, partial ²=0.025), suggesting comparable temporal IL-4 profiles between groups. Post-hoc pairwise comparisons between time points did not reveal any significant differences (all p&gt;0.05) (Figure 2).

Table 2 2

Salivary CXCL6

The mean values of CXCL6 in control and diabetic groups across the three time points are presented in Table 2. A two-way repeated-measures analysis was conducted to evaluate changes in salivary CXCL6 levels over time and between diabetic and non-diabetic groups. Mauchly's test indicated a violation of the sphericity assumption (p&lt;0.001); therefore, multivariate test statistics are reported. Multivariate analysis revealed a significant main effect of time on CXCL6 levels (Pillai's trace=0.243, F(2,24)=3.852, p=0.035, partial ²=0.243), indicating significant temporal changes following tooth extraction. Pairwise comparisons of estimated marginal means demonstrated a significant reduction in CXCL6 levels from baseline to 2 days post-extraction (p=0.009).

The between-subjects effect of group was not significant (F(1,25)=0.003, p=0.954), indicating no overall difference in CXCL6 concentrations between diabetic and non-diabetic participants when averaged across time points. Importantly, a significant time-by-group interaction was observed (Pillai's trace=0.231, F(2,24)=3.611, p=0.043, partial ²=0.231), demonstrating that the temporal pattern of CXCL6 expression differed between diabetic and non-diabetic groups. This interaction was further supported by a significant linear trend (F(1,25)=6.978, p=0.014), indicating distinct directional changes in CXCL6 levels following tooth extraction between groups (Figure 2).

## Discussion

In this study, we evaluated the early temporal dynamics of salivary IL-4 and CXCL6 following tooth extraction in individuals with and without diabetes, aiming to characterize acute wound-healing responses. IL-4 levels remained stable throughout the first 48 hours post-extraction, with no significant differences observed between groups. In contrast, CXCL6 showed significant time-dependent variation and a clear group-specific pattern, reflecting divergent inflammatory kinetics between diabetic and non-diabetic participants.

Tooth extraction creates an alveolar wound that heals through a well-described sequence of biological events, including inflammation, cell migration, proliferation, and tissue remodeling, similar to wound healing in other tissues (15). Diabetes mellitus is associated with systemic alterations that compromise these tightly regulated phases, with affected individuals frequently exhibiting prolonged inflammatory responses, impaired re-epithelialization, and altered immune cell function, ultimately contributing to delayed wound closure and increased susceptibility to infection (2). Together, these factors suggest that extraction socket healing represents a clinically relevant model for examining diabetes-related disturbances in early wound-healing dynamics.

A notable observation in the present study was the numerical differences in baseline salivary levels of IL-4 and CXCL6 between diabetic and non-diabetic participants, although these differences did not reach statistical significance. These baseline disparities remain biologically interesting, as they may reflect underlying differences in immune tone prior to surgical intervention. Several studies have reported lower IL-4 levels in saliva (16) and serum (17) in diabetic compared with healthy individuals. However, to date, no studies have evaluated salivary CXCL6 between healthy and diabetic individuals.

In our study, IL-4 did not exhibit significant temporal variation within the first 48 hours after tooth extraction in either diabetic or non-diabetic participants. IL-4 is a key anti-inflammatory cytokine that promotes alternative (M2) macrophage polarization, thereby supporting resolution of inflammation, tissue remodeling, and angiogenesis during wound healing (18). This action is consistent with IL-4's role in later stages of healing, where excessive inflammation has already been tempered and tissue regeneration is underway, rather than with the acute innate immune activation that dominates within the first hours after injury.

Chemokines and cytokines are pivotal in orchestrating wound healing events, with chemotactic signals guiding leukocyte infiltration and cytokines regulating the balance between pro-inflammatory and reparative responses. Chemokines, including CXCL family members play a central role in establishing chemotactic gradients that direct neutrophil and monocyte migration to the injury site, with these early cellular movements being essential for effective wound resolution and reconstruction of tissue architecture. Disruption of the timely regulation of these signals can delay healing or contribute to chronic inflammation, as observed in impaired healing conditions such as diabetes (3).

Our finding of a significant time-by-group interaction for CXCL6 indicates that the trajectory of this early inflammatory signal varies between diabetic and non-diabetic individuals and the overall CXCL6 levels declined over time in the pooled sample. This divergence in temporal patterns may reflect systemic and local immune alterations associated with diabetes, such as impaired leukocyte function, dysregulated chemokine gradients, endothelial dysfunction, and differences in microvascular responses. Diabetes is known to affect wound healing by disrupting multiple aspects of the healing cascade, including sustained or dysregulated inflammatory signals, delayed macrophage phenotype switching, and impaired angiogenesis, all of which can stem from hyperglycemia-induced metabolic stress and oxidative stress (19). Inadequate or improperly timed leukocyte recruitment leads to persistent inflammation and failure to progress appropriately into proliferation and remodeling stages, which has been documented in diabetic wound models (20). The rapid increase in CXCL6 from baseline to 1 hour post-extraction may reflect a compensatory acute inflammatory response in the diabetic group, potentially facilitating early leukocyte recruitment. Given the localized nature of extraction socket healing, future research may explore whether localized strategies aimed at regulating early chemokine signaling can influence inflammatory resolution and tissue repair in diabetic individuals. However, such approaches require careful evaluation of timing, dosage, and safety, as well as confirmation of causality.

The contrasting behavior of IL-4 and CXCL6 across the early post-extraction period underscores the complexity of immune regulation during wound healing. IL-4's role appears more relevant to later, anti-inflammatory and reparative processes, while CXCL6 reflects acute inflammatory and chemotactic activity that is sensitive to systemic context. Taken together, these findings emphasize that early healing responses involve a coordinated but distinct set of molecular signals, with some pathways remaining quiescent until later phases, and others responding rapidly to injury and systemic conditions. This insight supports the use of dynamic cytokine and chemokine profiling to characterize healing trajectories and local tissue response behavior.

Importantly, the divergence in CXCL6 behavior between groups occurred in the absence of a concomitant difference in mean levels averaged across time, reinforcing the value of analyzing temporal change patterns rather than static concentrations alone. In other words, the interaction effect suggests that the timing and pace of inflammatory modulation may be more informative about the healing trajectory than absolute biomarker levels at isolated time points. This perspective aligns with growing recognition in wound biology (21) that kinetic patterns of molecular signals, such as rise and resolution curves, are meaningful indicators of healing progression and may illuminate where the healing cascade deviates in pathological conditions.

A key clinical implication of these findings is that early post-injury measurements of specific chemokine dynamics may provide insight into tissue response behavior and healing readiness. For example, understanding whether a patient's chemokine trajectory does or does not align with expected inflammatory resolution patterns could inform risk stratification for delayed healing and related complications. While glucose testing remains the standard for diabetes detection and monitoring, dynamic profiling of healing-related cytokines and chemokines could complement clinical assessments by identifying aberrant early healing responses that warrant closer observation or intervention.

This study has several important limitations that warrant consideration when interpreting the findings. First, the sample size, particularly in the control group, was relatively small, which reduces statistical power and limits the ability to detect subtle group differences in cytokine dynamics. Saliva-based cytokine measurements also introduce inherent variability, as salivary concentrations are influenced by flow rate, circadian rhythms, hydration status, oral hygiene, local inflammation, and microbial composition. These factors may obscure systemic differences and complicate the interpretation of cytokines such as IL-4 and CXCL6, which exert both local and systemic regulatory roles.

Another limitation is the narrow postoperative assessment window used. Cytokine dynamics evolve rapidly, and changes beyond the first 48 hours may reveal group differences that were not apparent in the early inflammatory phase. Furthermore, only patients with well-controlled diabetes were included, which limits generalizability to individuals with moderate or poor glycemic control, where more pronounced inflammatory alterations might be expected. Overall, future studies incorporating larger and more diverse cohorts, longer follow-up periods, and broader biomarker panels will be essential to clarify the mechanisms by which diabetes influences oral wound healing.

## Conclusions

Early post-extraction healing in individuals with diabetes is characterized by distinct temporal inflammatory responses. Salivary IL-4 levels remained stable during the first 48 hours after tooth extraction, consistent with its role in later reparative phases. In contrast, CXCL6 showed significant time-dependent and group-specific modulation, reflecting altered early inflammatory kinetics compared with non-diabetic individuals.

## Figures and Tables

**Table 1:** Table Demographic characteristics of the study population.

Variable	Diabetic (n=20)	Control (n=10)	pvalue
Age (years), mean±SD	57.95±7.48	35.2±7.58	0.066
Gender (M/F)	16/4	6/4	0.384
HbA1c (%)	6.950±0.2351	4.750+.3629	.227
Random glucose (mg/dL)	138.40±14.069	113.70±13.825	.825

1

**Table 2:** Table IL-4 and CXCL6 levels in diabetic and healthy control groups at baseline, 1 hour and 2 days following tooth extraction.

	Sample number	Baseline level. Mean±Std. Deviation. Pg/ mL	One hour after extraction. Mean±Std. Deviation. Pg/ mL	Two days after extraction. Mean±Std. Deviation. Pg/ mL
IL-4 Control	10	202.40±47.47	191.36±83.14	191.10±103.61
IL-4 Diabetic	20	177.24±72.07	138.28±53.54	191.46±97.06
CXCL6 Control	10	967.65±917.88	446.09±3 69.03	321.39±137.40
CXCL6 Diabetic	20	491.72±616.37	839.93±2248.44	469.58±590.25

2

## Data Availability

Declared none.
